# Correspondence

**Published:** 1914-05

**Authors:** John D. Bonnar


Yours very truly,

GEORGE H. NORTON,

Deputy Engineer Commissioner.

---

March 23d, 1914.

George H. Norton, Esq.,
Deputy Engineer Commissioner,
Buffalo, N. Y.

Dear Sir:—

In replying to your communication of the 2d. inst. I would do so in a wholly individual capacity, as we assume the questions propounded in your letter, are such as all medical men are fairly familiar with, and would gladly answer, were such directed to them. That the lot has fallen upon me, while appreciated, is also accepted with full knowledge of its responsibility.

Your paper, presented to the Medical Society of Erie County, on the 16th ultimo, upon the subject of "Water and Sewerage Systems of Buffalo," which you say "opened a sharp discussion," on the part of the doctors present, but "fell short of your expectation in answering the question, "Why you cannot with equal expenditure save at least two lives from these other diseases—diptheria, syphilis, whooping-cough, and tuberculosis, to one from typhoid by water filtration?" (I assume you meant to include also scarlet fever and measles).

As a civil engineer, your service deals with non-vital, material bodies, while physicians, in their service, deal with vital physical bodies. Yours, constructive, ours, instructive; yours physical, ours, chemical. Thus in the matter of health, the tangents of our spheres of influence, approach each other in the matter of public sanitation.

Needless to say, we are, as citizens, alike related to the public welfare, but in professional work, meet only on sanitation of water and sewer systems.

Typhoid fever, being a germ disease, mostly transmitted through water, affords that common ground, upon which the skill of the physician and the constructive technique of the engineer are needed to secure the fullest efficiency.

The other infectious diseases, above mentioned, being mostly transmitted through the air—save venerial—present scientific problems, which, you will readily appreciate, must be met entirely by the physician, without any possible assistance from the engineer.

According to statistics, upon the death-rate in Buffalo, we find the following significant figures:—

| | In 1882 | In 1913 |
|---|---|---|
| Buffalo's Population | 175,000 | 450,000 |
| Deaths From: | | |
| Scarlet Fever | 329 | 15 |
| Diptheria | 290 | 41 |
| Whooping-Cough | 54 | 30 |
| Measles | 85 | 68 |
| Pulmonary Tuberculosis | 356 | 566 |
| Typhoid | 121 | 68 |

The contrasts are so conclusive that no argument is needed to establish the proof of our having reached and even greatly exceeded the quite arbitrary demand of saving "with equal expenditure—at least two lives from these other diseases, to one from Typhoid.'

After the expenditure of some five million dollars for water purification, we find the effectual means in the cheap "Chlori hation" process, which is chemical and bacterio-logical and not an engineering problem.

With but about one fourth of a million of dollars of public expenditure for hospitals for the care of all the other diseases mentioned, than typhoid, the foregoing cited decline in death-rate, make further comment needless.

Coupled with these is practical immunization, by means of typhoid vaccination, as proven by results in U. S. army, as applied by Surgeon-General Dr. O'Riley, the fruit of labors by such physicians as Ehrlich, Wright and Pasteur, in quest of scientific means of meeting and conquering human disease.

With the expenditure of but a fraction (not the "equal" amount you suggest) for segregation hospitals, a practical elimination of the infectious diseases would be possible.

You must certainly agree that even within the short confines of a letter, your desire of being shown, is fully met by the foregoing facts.

Yours very truly,

JOHN D. BONNAR, M. D.

